# Keeping the culture alive: the laboratory technician in mid-twentieth-century British medical research

**DOI:** 10.1098/rsnr.2007.0035

**Published:** 2008-01-09

**Authors:** E.M. Tansey

**Affiliations:** Wellcome Trust Centre for the History of MedicineUniversity College London, 183 Euston Road, London NW1 2BE, UK

**Keywords:** medical laboratory technicians, medical laboratories, medical history

## Abstract

This paper reports results from a detailed study of the careers of laboratory technicians in British medical research. Technicians and their contributions are very frequently missing from accounts of modern medicine, and this project is an attempt to correct that absence. The present paper focuses almost entirely on the Medical Research Council's National Institute for Medical Research in North London, from the first proposal of such a body in 1913 until the mid 1960s. The principal sources of information have been technical staff themselves, largely as recorded in an extensive series of oral history interviews. These have covered a wide range of issues and provide valuable perspectives about technicians' backgrounds and working lives.

## Introduction

Recent perspectives from patients, nurses, unorthodox practitioners and others associated with medicine and health care have provided valuable contrasts to analytical accounts of more conventional medical practice. These historiographical movements have greatly enhanced, and extended, our understanding of the development, diversity and impact of medicine. During the twentieth century ‘scientific medicine’ has also become more diversified and specialized, and professional medical research has encompassed a varied array of personnel.^1^ These developments have been synergistic: as laboratories have acquired specialized equipment and developed new techniques, they have required a group of specialized personnel to build, maintain and operate apparatus, and as these new kinds of staff have entered labs they have in turn contributed to the development of new equipment and techniques.

It is now timely to recognize and consider historically the roles of some of these other players who are often seen as minor, especially as the importance of teams rather than individuals in research is being increasingly recognized.^2^ Foremost among these ‘other players’ are, and for over a century have been, the laboratory technicians. This paper reports on a historical project to investigate the working practices and contributions to medical research of technical staff who are intimately involved with laboratory procedures and developments. Technicians have played, and continue to play, key roles in both the routine maintenance and innovative research work of a laboratory and are extremely important in the training, both implicit and explicit, of new generations of scientists. They are crucial in passing on knowledge, practical skills, attitudes and approaches; metaphorically—and quite literally in some circumstances—they keep alive the very ‘culture’ of a laboratory. Sociological analysis, especially the interview and observational studies by Bechky and Barley of technicians in Cornell University's Biotechnology Centre in the early 1990s, has provided some useful models and concepts with which to examine technical work.^3^ However, these activities have not, as yet, been accorded a great deal of documentary or analytical attention by historians.^4^

By and large, the modern laboratory technician has evolved from the tradition of personal laboratory servants, assistants and amanuenses employed in the laboratories of seventeenth-century natural scientists. This tradition persisted into the early years of the twentieth century, as scientists such as Charles Sherrington, at St Thomas' Hospital in London, and J. J. Thomson, at the Cavendish Laboratory in Cambridge, employed ‘lab boys’ who became indispensable assistants. Later, unqualified assistants began appearing more routinely in labs, the ‘qualifications’ for the job being skill and experience. After World War II normal training courses and recognized qualifications were developed, and in some circumstances the technician became a collaborator and partner in research. In recent years the specialist skilled technician has undergone a further transformation, and graduate and postdoctoral technicians can now be found occupying such posts for short periods as a step to another professional appointment, rather than as a career in itself.^5^

This preliminary examination will concentrate on laboratory technicians working for the Medical Research Council (MRC) in twentieth-century Britain, especially at the National Institute for Medical Research (NIMR). It is part of a larger survey of medical laboratories in research, teaching, healthcare provision and industry. The purpose of this research has been to find out who technicians were, what kinds of jobs they were employed to do, what training they received and how specialized skills and training have been cultivated and evaluated, and how technical careers developed; and to try to chart the changing roles and contributions that technical staff have contributed to routine management and experimental investigations in British medical research laboratories from about the time of World War I to the late 1960s. Some assessments will also be made of how technical staff were perceived within the hierarchy of medicine and medical research by the professional scientists and administrators for, or with, whom they worked. As such it is part of a much larger examination of the internal history of medical science in twentieth-century Britain.

## Finding and recording technicians

Technicians have proved a difficult group to trace historically. They rarely leave material records in archives, write reminiscences or receive obituaries. They can be troublesome to track via published staff lists in organizations that equate the word ‘staff’ with ‘academically qualified scientist’, and are frequently not even acknowledged in the scientific papers to which they contribute, let alone credited as co-authors. The material used here, relating to the NIMR, has been largely derived from technicians' or retired technicians' own accounts of their working lives obtained from extensive oral-history interviews.^6^ Interviewees were found by using advertisements in local newsletters, from recruitment at a Pension Club meeting and by word of mouth. This material has, *inter alia*, revealed details of their backgrounds, training activities, pay and conditions, the impact of professional trade-union associations, and formal training and accreditation procedures. Where possible these have been supplemented by relevant official records, and obituaries and reminiscences of scientists who either started their careers as laboratory technicians or describe their assistants and staff.

## Before 1920

Before World War I the routine employment of assistant staff was uncommon in the few laboratories associated with medical research. Occasionally young ‘lab boys’ fresh from school were paid for personally by the man for whom they worked, and they were gradually trained as that person's bespoke right-hand man. The gradation from boy to technician can be demonstrated by the career of George Cox, originally employed by the future Nobel Laureate physiologist Charles Sherrington when he started his independent professional career at St Thomas' Hospital, London, in 1887. Cox followed Sherrington to Liverpool in 1895 and thence to Oxford in 1913. By then Cox was recognized as a ‘technician’, a man with a wide array of skills essential for the laboratory—he assisted with surgical operations, developed and maintained equipment, and performed essential tasks such as chemical analyses or histological sectioning, and was himself in charge of a team of five or six ‘boys’.^7^ At the Cavendish Laboratory in Cambridge, ‘Ebenezer’ started work for J. J. Thomson in 1886. He gradually became invaluable: assisting at experiments and directing other assistants, and, as the fourth Lord Rayleigh reported, he was then accorded the title ‘Everett’.^8^

What, then, did these ‘boys’ do? One such, T. J. Surman, joined the lab as a 14-year-old in 1918.^9^ Initially he thought that his duties were ‘doing anything that anybody else in the lab told me to do’, although he soon settled into the routine of helping Cox lay out the equipment and instruments for Sherrington's research. Cox gave Surman guidance on the care of animals and use of equipment, but it was Sherrington himself who taught the young man the operative and experimental techniques that he was then writing up in *Mammalian physiology, exercises in experimental physiology*, a book that influenced generations of physiologists around the world. As the most junior member of the laboratory, Surman was given particular responsibility for looking after the experimental animals. This meant that on Sunday, the only non-working day of the laboratory, he was expected to feed and water the animals, and during the annual two-week closure of the department in August, when everyone took their holidays, he was still required to go into the lab every day to tend the animals.

A somewhat different situation evolved in the Wellcome Physiological Research Laboratories (WPRL), private laboratories owned by the pharmaceutical manufacturer Henry Wellcome. Established as a small laboratory facility in central London in 1894, to manufacture the new ‘wonder drugs’ of serum anti-toxins against diphtheria and tetanus, this institute expanded to an estate in Herne Hill, South London, in 1899. Expansion of scale was accompanied by diversification of production, and experimental work was started to produce other anti-toxins and develop a programme of physiological research into the effects of natural chemical products. The then Director, Dr Walter Dowson, instituted a regular policy of employing assistants by contacting Dr Baker, the headmaster of the local Alleyn's School, an offshoot of Dulwich College, with the intention of employing boys with a good science background at the WPRL.^10^ The first six schoolboys were recruited in 1899. One of them, A. T. Glenny,^11^ joined Dowson in the bacteriological laboratory, and another, A. J. Ewins, worked in the chemical laboratory.^12^ Dowson also routinely assigned these boys to other sections of the WPRL, to learn a wide array of techniques and methods. These positions were clearly different from the other ‘assistant’ positions at the WPRL, which included a general laboratory assistant who cleaned and fetched and carried, another assistant who prepared nutritive media for the bacteriological production, and several ancillary workers who either looked after animals or bottled and labelled the sera.

The situation at the WPRL can be seen as incorporating elements of not only the ‘servant’ model already present in some laboratories, but also the apprentice model used in trade and industry with which Henry Wellcome, as an industrialist, was more familiar. In such a scheme, these boys received a sound training in the practical skills necessary for precise jobs. Young men such as Glenny and Ewins were recruited to learn higher-grade skills than the lab ‘boys’ also employed in the WPRL. They were trained to assist with innovative research in specific capacities and were all encouraged to develop their technical and, most unusually, academic skills. Glenny and Ewins both studied for external University of London degrees, Ewins in chemistry, Glenny in mathematics. Thus qualified, they were then appointed to the scientific staff of the WPRL, and both became successful professional scientists and Fellows of the Royal Society. Ewins left the WPRL in 1914 with the then Director Henry Dale and the chemist George Barger to form the Department of Biochemistry and Pharmacology at the NIMR. From there he moved to May & Baker in 1917 as Director of Research, and it was under his direction that the anti-streptococcal agent ‘M&B693’, famous for ‘curing’ Winston Churchill of pneumonia during World War II, was developed.^13^ In contrast, Glenny spent his entire career at the WPRL, contributing substantially to research on bacteriology and immunology, and taking charge of the serum-producing activities. One of his major contributions was to devise a ‘patient record card’ for each horse, on which were recorded all injections, bleedings and daily temperatures, as a guide to the progress of infection. These meant that full bleedings, from which the therapeutic serum was manufactured, could be made when anti-toxin titres were highest, thus increasing the production of saleable serum. Using these cards, Glenny boasted that he could retrieve information about any horse in less than 90 seconds, and during World War II he controlled more than 1500 horses with this system.^14^

Both Glenny and Ewins became Fellows of the Royal Society when their former Director, Henry Dale, was President. Their success must have been particularly rewarding to Dale, because one of his first tasks, when he became Director of the WPRL in 1904, had been to address the careers of all these young men recruited by his predecessor. He retained only Glenny and Ewins and those found wanting were rapidly advised to find alternative careers.^15^ When Dale left the WPRL in 1914, it was to join the staff as one of four departmental heads of the MRC's proposed new research institute, and during much of the rest of his career he was to become further involved in the development of technical training.

## The MRC and the NIMR

The institutional focus of this paper is the principal research lab of the MRC (created as the Medical Research Committee in 1913, becoming Council in 1920; MRC is used for both). At its very first meeting the Committee considered it desirable to have its own ‘Central Research Institute’ and began to recruit staff. World events overtook these plans, and what staff had been appointed by August 1914 had to work in temporary accommodation until 1919, when the NIMR was opened at the former Mount Vernon Hospital in Hampstead, North London. It remained there, with a separate farm laboratory in Mill Hill, North London, for 30 years, when most of the laboratories relocated to a purpose-built institute adjoining the farm in 1949.

The necessity for laboratory attendants to assist scientific staff was accepted without question by the MRC from the very beginning, and during World War I the minutes of the Council's meetings record occasional appointments, each made on individual application by a particular scientist, for the appointment of a named assistant. However, unlike appointment to the scientific staff, no pension allowance was made for these employees. By the time the NIMR became a physical reality in 1919, there were several members of the ancillary staff. Each of the four departmental heads in applied physiology, biochemistry, bacteriology and statistics had a personal technician to assist with their own experimental work. Other scientific staff were not provided with technical assistance, although sometimes they might ‘share’ the head of department's technician. There were also two or three boys or junior staff, and some maintenance and animal house staff, many of whom were ex-servicemen.

## How were technicians recruited?

Few of those who became technicians at the NIMR before World War II had a clear idea of what the job entailed. Most were grammar-school boys from North London, often with a declared interest in science or in ‘how things work’. Some just wanted a job. As Den Busby, who came from Hampstead and started work at the NIMR in 1934, recalled, ‘… we weren't looking for jobs as technicians. We were looking for work of some kind, because this was in the thirties, and the Depression was just, sort of, tailing off at that point’.^16^ The NIMR, a large local employer, was the obvious place to try. Arthur Hemmings went to the NIMR in 1932, only the second secondary school boy to be employed there.^17^ He was known to Sayers, the NIMR's administrator, and taken on immediately after he applied. Apart from a short break working at University College Hospital, London, in the mid 1940s, Hemmings was at the NIMR until his retirement in 1976.

*Ad hoc* school recruitment continued after World War II, although by then some local science teachers and employment advisers specifically directed scientifically inclined school leavers towards a career as a lab technician. One was John Clark in 1954, who noted: ‘my school put me in touch with the Youth Employment Agency. I thought that I'd like to work in science. … The Agency agreed and arranged an interview at NIMR. … I was good at biology, so I wrote to the MRC at Mill Hill, and said, “Have you got a job?” And they said, “Come along and see us.” And I did. When I was offered the job I accepted.’^18^ As with many others it was the first, and sometimes the only, job he ever applied for.^19^

Others found it more competitive. Just five years earlier, Alan Brownstone applied to the NIMR after starting but not completing a chemistry course at the Battersea Polytechnic after his National Service. He recalls that about 30 people applied when he did, and when he was offered and accepted a position he expected to stay for a year or so, before moving on to full-time study. In the event he retired from the NIMR 39 years later.^20^

## Pay and conditions

It was at the beginning of 1918 that Henry Dale, as head of the Department of Biochemistry, and Leonard Hill, head of Applied Physiology, wrote directly to the MRC about the terms and conditions offered to technical staff. Each had a personal technician, Mr Starling and Mr Webster respectively, who, like Dale and Hill themselves and all other MRC employees, held individual contracts directly with the Medical Research Committee. There was no general salary scale in operation, and Dale and Hill approached the MRC to argue separately for individual pay rises for their own technicians. These were approved, but on an *ad hominem* basis.^21^

As the NIMR's programme of work got under way, the MRC realized that the system of individual negotiation with, or on behalf of, each member of staff would soon become unworkable. As early as 1920 a formal scale determining pay and pension provision for *all* their staff—technical, maintenance and scientific—was issued for the first time. This showed a considerable degree of foresight and intention: at the time the MRC employed only nine scientists and about 15 assistants at various levels.^22^

Dale was adamant from the very beginning that the MRC should employ good technical assistants, and he raised the issue explicitly in 1921 with the Secretary of the MRC, Walter Morley Fletcher. When Director of the WPRL, Dale had been used to high-grade assistance by highly trained technicians of the calibre of Alexander Glenny and Arthur Ewins, in addition to having general support in the routine work of the laboratories. He emphasized to Fletcher that a dedicated research institute such as the NIMR did not have ready recourse, as did a university department, to a pool of students who could assist at experiments as part of their training. Dale reflected on his own wartime experiences when, newly employed by the MRC, he had worked in temporary accommodation at the Lister Institute. He stressed to Fletcher that ‘the results of some years of experience at the Lister, is that the efficiency of its salaried staff is seriously impaired by the fewness and inefficiency of the laboratory attendants’.^23^ The MRC apparently took notice, because a few more assistants were gradually added to the staff, such that by 1926 Dale made a further recommendation that a higher grade of ‘technical assistant’ or A grade be created, for particularly designated staff, who would receive a higher salary and superannuation provision.^24^ Precisely how many additional assistants were appointed after this time is not clear, although one measure can be garnered from the reminiscences of Len Ward, who was appointed as a lab boy in 1928. He estimated that at that time the complement was 7 or 8 A men, 10–12 B men and 3 or 4 boys, plus 6–10 general maintenance, boiler house and animal farm staff, figures that suggest an increase in staff numbers of between 70% and 100% since 1920.^25^

Further negotiations and administrative rationalizations throughout the 1920s meant that by the end of that decade there was a proper scale not only for pay but also for promotion, and a ‘profession’ that was seen as a career option for bright young school leavers was beginning to be recognized; nevertheless even by the end of the 1920s ‘boys’ were still employed to fetch, carry, mend broken apparatus, and help in the manufacture of new equipment. When Len Ward started in the animal house in 1928 his job was to care for the rabbits and chickens, preparing their feed, cleaning out the cages and burning all the resultant waste on what he has described as an ‘everlasting’ bonfire in the NIMR's grounds at Hampstead. He was so small he had to stand on a box to reach the highest cages, apparently causing much amusement to his bosses in so doing.^26^ When asked what his duties were, he replied:

Everything. I was the lab boy. I held the animals when they wanted animals injected, I made up any solutions they wanted, I helped to make apparatus that was required, I did almost everything for them. All the services and things that were there. And then in my spare time, I used to dust the lab, every morning, the lab had to be dusted every morning. And once a week all the shelves had to be cleared and bottles taken off.^27^

Moving up the pay scale could be difficult. Arthur Hemmings recalled that when he joined in 1932, a B-grade technician aged 21 years received 50 shillings per week, with the possibility of a rise of 2 shillings per week per year until 70 shillings a week was reached at the age of 30 years. That was the maximum, and staff could be stuck at that point for many years. By the end of the 1930s there was growing resentment at the NIMR as B technicians were effectively waiting for dead men's shoes for promotion into the restricted A grade. Not surprisingly, A-grade staff were content with the situation and wanted no change, and in Dale the B technicians had, by then, a Director who was either unsympathetic to their aspirations or so heavily burdened with other duties that this was a low priority.^28^ His successor in 1942 as Director, Sir Charles Harington, and the times, in the middle of a war that had recognized and valued technical skills and experience, proved more amenable. After much internal negotiation led by two B technicians, and with advice from the Association of Scientific Workers (AScW) who used a pharmaceutical company's pay scales for technicians as an explicit comparator, a more equitable pay scale was established, with clear intermediate points for the so-called A/B grade.^29^ That pay scale came into operation in 1944, and allowed at least one young technician, Arthur Hemmings, to get married. Hemmings had said that he would not get married on less than £5 a week. The new pay scales gave him £5 1s. 9d.^30^ However, almost immediately after the end of World War II, further agitations and discontents about technical pay scales became obvious.

The AScW had a prominent role in assessing salaries for technical and/or junior scientific staff across a range of scientific specialities, often in association with another, relevant union. Thus, for example, pay scales in the engineering industry were assessed jointly with the Engineering and Allied Employer's National Federation in 1946,^31^ although a review of the drug and fine chemical industry in the following year was done solely by the AScW.^32^ Overshadowing many of these discussions and debates was the creation of the National Health Service (NHS) and the need to establish appropriate union representation and negotiation machinery. Although these movements are outside the scope of the present paper, of particular relevance was the effect of the NHS on staff in medical schools and the MRC, who were not included in such arrangements.^33^ However, the impact of the NHS and the development of negotiated pay agreements for its staff, including technicians, could not be ignored by the MRC. In April 1949 medically qualified scientists employed by the MRC were given parity with similar staff in the NHS. Other staff, non-medically qualified scientists and technicians could not, the MRC claimed, be so rewarded without the specific permission of the Treasury, which had not been forthcoming. The AScW mounted a concerted campaign to attract members of MRC staff, and many technicians and non-medically qualified scientific staff at the NIMR hurriedly joined the union, which then made formal representation on their behalf to Government for comparability with the NHS.^34^ However, Union meetings were strictly banned from NIMR premises, and local pubs and school and church halls were used as meeting places until formal recognition was agreed by the MRC.^35^ An agreement was finally approved by the Treasury towards the end of 1950. The AScW's published report notes somewhat laconically that ‘throughout the “dispute” relations between the Association and the Council of the MRC have been most cordial and it is not too much to hope that in the future we shall be able to meet the MRC *before* difficulties arise’.^36^

Before World War II technical training seemed to be offered on a rather *ad hoc* basis. Individual scientists trained ‘their’ technicians to do the precise work they required, and in larger divisions a technician might rotate through several positions and gain a wider experience, but there was no fixed pattern. Increasingly it was the technicians themselves who recognized the limitations of their training, and many found night classes to go to, at their own expense, to improve their skills and increase their ‘saleability’ not only within the internal NIMR/MRC marketplace, but also externally. Immediately after the war several junior technicians were studying a biology evening course at Harrow Technical College when, to stem falling numbers, the college authorities decided to change it to a day course. This caused an immediate problem, and a small delegation of technicians approached the then NIMR Director, Sir Charles Harington, to ask for official time off during the day to enable technicians to continue their studies. Harington steadfastly refused to consider such a request. Day release or time off in lieu was beginning to be an accepted part of technical training in related industries, and in a 1947 review by the AScW, many companies, including the pharmaceutical firm May & Baker, were commended for allowing junior staff study leave.^37^ After considerable negotiation, Harington finally relented, and after formal sanction from the MRC, day release was allowed for approved technicians on individual application.

Because he had risen through the ranks and become more skilled himself, often as a result of taking night classes, Arthur Hemmings became particularly concerned at the somewhat piecemeal arrangements for technical training. After the war he started to organize in-house training courses, at which senior technical staff, and sometimes the scientists, would lecture on theory and demonstrate basic techniques to groups of junior technicians.^38^ By the early 1960s, however, entry qualifications had become more rigorous and a basic training course for technicians was deemed unnecessary, although specialized courses in the animal house and general workshop were continued. Jon Marsh, for example, joined the NIMR in 1960 in a cohort of other young school leavers who all became junior technicians together. He had nine ‘O’ levels, and was awaiting the results of three science ‘A’ levels. Once he had those he was promoted to Junior Technical Officer and immediately given day release to study applied biology at Brunel College.^39^

In February 1946 three organizations, the Association of Scientific Workers, the Association of University Teachers and the British Association of Chemists, convened a meeting to discuss ‘The problem of training laboratory technicians’.^40^ Acknowledging that there was no coordination between various courses available to technicians, and that in some fields there were no suitable courses at all, the conference, attended by 110 people, recognized that there was an urgent need to address the issue of technician training. Proposals for a national scheme of training and professional certification, supplemented with further specialized advanced qualifications, were supported, and a committee was nominated to address the practical issues associated with implementing such a request. Relevant organizations and institutions then became involved in devising and implementing such courses, both locally and nationally.

By the beginning of the 1960s there was not only encouragement, but also the clear expectation, that NIMR technicians would attend one of these approved training courses, offered either internally or externally, at appropriate stages in their careers. Pamela Bradburne, who started at the Common Cold Research Unit in Wiltshire, an administrative part of the NIMR, explained her introduction to work: ‘You had to go one night a week to the Path[ology] Lab in Salisbury Infirmary; and there we used to learn Bacteriology, Biochemistry … and everything else. And of course the virology bit was done at the Common Cold Unit. So we went through the basic path lab training and ended up with the Certificate for Intermediate Laboratory Technology.’^41^ After this academic training, and general lab experience in the Unit, technicians would be allocated to specific projects, where Pamela Bradburne was astonished that eminent scientists explained things to her: ‘these very clever people bothering with the likes of us. … But we did go to seminars and things. We were expected to go as Technicians.’^42^

## The status of technicians in the NIMR

Den Busby has graphically described the situation he found at the NIMR when, aged 15 years, he started work there in 1934:

there were very acute divisions, between Technical Staff [and] Scientific Staff. Because in the first place, we were Assistants, or Lab Boys. We weren't Technicians: that word hadn't been invented. The scientists were mainly BScs and Doctors of Medicine, that sort of thing. And they were an entirely different kettle of fish. I mean, if the Senior Scientists were in the lift, you weren't allowed to use the lift. And if you did get into it, with a bucket or something, which you were allowed to take up in the lift, and, [if] for example Sir Henry Dale was in the lift, well then you got out again, you see; and then you called it back and used it when it was empty.^43^

Even 15 years later, Busby, then aged 30 years, was referred to by his boss (Sir) Christopher Andrewes as his ‘lab-boy’. ‘It didn't worry me’, he recalled, ‘but I've always remembered that. I thought, ‘Good Lord! I'm still only his lab-boy!’^44^

There were some quite explicit differentials. All the technical staff, for example, wore brown lab coats, whereas the scientific staff had white coats. Alan Brownstone recalled:

Harington was … Edwardian in his outlook, if you like; I mean when I first went there the technicians all wore brown coats, and it was quite a decision for him to make his mind up that we should be allowed to wear white coats. The comment was, ‘Well who the hell's going to know the difference between a Technician and a Scientific Staff, if the Technicians aren't wearing brown coats?’^45^

Curiously, however, women were exempt from this colour-coding differentiation, and the few women at the NIMR, whether scientists or technicians, all wore white coats. Perhaps women had been so rare that no consideration was given to the question of coat colour until the precedent that all women wore white coats had been set. Or as women could be clearly recognized as distinct (and perhaps inferior?), there was no further need for different grades to be distinguished.^46^

## World War II

World War II had an impact on the lives and careers of technicians from the NIMR in many ways. Of considerable significance was that several were called up to serve in the armed forces, their specialized technical skills being readily used and recognized as providing and supporting essential services. This reinforced, or even created in some individuals, a new and palpable sense of importance and worthiness. Many were employed around the country in establishing blood transfusion and public health labs, often in positions of responsibility and authority that they had not been in before. Others served abroad in military hospitals and public health units, and in due course many returned to the NIMR with new skills and new attitudes.

Simultaneously, when faced with a haemorrhage of trained people, the MRC explicitly acknowledged the importance of technically skilled staff by arguing at tribunals, usually successfully, for their exemption from conscription on the basis of their unique value as doing work of national importance.^47^ These factors all reinforced many technicians’ sense of purpose and worth. Some unusual situations arose as a consequence, as epitomized by an experience of Den Busby. In 1944 he was serving in the Royal Navy when his boss at the NIMR wanted him back in London. Busby recalls, ‘Sir Christopher Andrewes applied to the Navy for me to be released, you see, which they wouldn't do. So they seconded me to the MRC. So I received a draft chit to “HMS MRC”, with Andrewes as my Commanding Officer.’^48^

All staff who remained in Hampstead found themselves undertaking new responsibilities as the Institute faced fresh emergencies. These included: serving in ARP (Air Raid Precautions); being members of an Institute-based auxiliary of the Hampstead branch of the London Fire Brigade; and being on nightly fire-watch in the Institute. The contacts and experiences instigated by several of these shared duties gradually eroded some of the prewar social barriers that had existed between different grades of staff.^49^ Many of the interviewees, for example, have commented on the camaraderie induced by fire-watching. One simple illustration will suffice: use of the Institute tennis court had been strictly segregated before the war—scientific staff could book the court on Monday, Wednesday, Friday and Sunday, whereas ‘assistant staff’ were allowed Tuesday, Thursday and Saturday afternoon.^50^ This rapidly broke down during the war, as sets were made up of available players regardless of rank.

Not all staff were comfortable with the emerging new order. Den Busby was equivocal about the changes, commenting:

I think it had changed in the sense that people started becoming much more Christian-name. It was something I could never really take to. I could never adapt to that, I suppose, having been brought up in this strict regime. I do remember once calling Dr Porterfield ‘James’, and I felt quite staggered at my temerity.^51^

## After the war

Several postwar changes affected the technical staff of the NIMR. The experiences and changed attitudes of both the returning and remaining staff, the move to a new, larger, purpose-built institute in 1949/50, and the arrival of Nobel Laureate Sir Peter Medawar in 1962 as Director, all contributed to massive changes. Medawar's arrival was particularly notable because he took a much more relaxed attitude to hierarchies and discipline than had his predecessors. According to Gill Ostler:

Sir Peter Medawar was like a breath of fresh air. He came in with new ideas; he was very, very dynamic. He had a tremendous personality. He was a person who communicated with everybody. And he brought his staff with him. And his group were— there seemed to be no demarcation lines as far as they were concerned: they were all friends together; they played cricket together—Technical Staff, Scientific Staff, PhD students—it was just one big group. And this really affected the Institute. And one can see, really, when Sir Peter Medawar came, he sort of broke down the barriers.^52^

The separate dining rooms (see below) were all amalgamated into one large area in a new extension to the Institute building; the hated ‘signing-in’ book was abandoned, and a five-day week became the norm for all workers. Alan Brownstone agreed:

When Medawar took over there was a very distinct change in the disciplinary attitude of the hierarchy, if you like to put it that way. I mean, one of the things, you see: we had to sign in the mornings when we came in, and the books were left out until I think about five minutes after the time you had to be there, about nine o'clock. And if you hadn't signed in there was a late book; and if you signed in the late book more than about three or four times a month you got called in front of the Head of Division. And if you kept on doing it you got called in front of the Director; and if you kept on doing it you got the push. It didn't matter how late at night you'd stayed, which was one of the things that used to annoy me. And … that was the sort of thing. But when Medawar came there was an immediate— I mean he came with— he brought Avrion Mitchison with him, who set up a Division, and he was very relaxed to put it mildly. He used to walk round in shirt sleeves and no tie, and carpet slippers. And he— I mean his people, they weren't going to sign in, *full stop*, and none of this blinking nonsense about signing in the morning.^53^

## Women technicians

There were very few women technicians before World War II. Arthur Hemmings has commented that ‘it was very male dominated’, and could recall only two women during his early years at the NIMR: Miss Cooks who became professionally qualified, and Sadie Carswell, a botany graduate unable to get another job who was (Sir) Alan Parkes's technician.^54^ As experienced men were conscripted for their technical skills and expertise during World War II, more women began to enter the labs to replace them. One later became Mrs Arthur Hemmings. Other women in the Institute included ‘a couple of women in the animal house’, Miss Baverstock the Librarian, two or three secretaries, and Mrs Cutts, who was Sir Henry Dale's secretary. In the women's dining room at Hampstead, there was a clear hierarchy and each senior woman had her own chair. ‘My wife’, added Hemmings, ‘used to get out of there as quickly as she could’.^55^

As was usual in many organizations at the time, all women staff, scientific as well as technical, were paid less than their male colleagues. Reviews after World War II by the AScW of technical salary scales in, *inter alia*, the pharmaceutical and chemical industries, in the health service, and in the MRC, recommended (albeit as a footnote) that ‘All scales should be irrespective of sex’.^56^ There were a number of other distinctions. Alan Brownstone recalled that Sir Charles Harington would not allow women to wear trousers, and would grant permission only in the very harshest winter conditions.^57^ At the same time, he had the discretion to allow ‘a member of staff with heavy domestic responsibilities, e.g. a married woman with children, or a person who lives alone and undertakes her own household duties’ reasonable time off work for shopping.^58^ Another technician, Mrs Julie Altringham, also remembered Harington's dislike of married couples working in the same lab (which was how she had met her future husband) and that they had needed ‘some sort of dispensation’ to continue to work together after marrying.^59^

## Domestic and social arrangements

At the NIMR in Hampstead there were a series of different dining rooms. The scientific staff, junior technicians, senior technicians, and women of all ranks all ate in separate small rooms. After the move of the NIMR from Hampstead to Mill Hill in 1949/50 the inefficiency and the physical inconvenience for the kitchen staff in servicing four different areas was increasingly recognized, and Peter Medawar created one central dining area on the ground floor in a new extension at the back of the building, although not without some grumbling discontent. Even then, many of the earlier distinctions were maintained; as Arthur Hemmings recalled, ‘the secretaries would sit together, the technicians sat with their friends, it was very cliquey’.^60^ In addition to the new dining room, several smaller rooms were added: ‘they had … social rooms put in as well, with television and a little library, and as I say, the billiard room and table tennis and that sort of thing which hadn't existed upstairs’.^61^ This social club, called NIMROD, also became involved in running the small library that technicians had started before the war. This library, of technical and reference books to assist those taking additional courses, had been run entirely by technical staff for technicians. The library relied very heavily on donations of books, often from scientific colleagues, and also held dances and other social events to raise book-purchasing funds.^62^

## Acknowledgements and authorship

It was, on the whole, unusual for technicians to get their names on publications, however large their contributions, before World War II. In July 1940 Henry Dale wrote to Edward Mellanby, Secretary of the MRC, seeking explicit clarification on the issue.^63^ The need for such clarification was largely because of the somewhat administratively awkward situation in which Mellanby also ran a Nutrition Research Laboratory within the NIMR, and Dale did not wish to be seen encouraging or discouraging a practice that might be common in the Secretary's laboratory. ‘It is obviously important’, he wrote, ‘that our practice should conform with your own at the Nutrition lab.’ Dale reported that at the Lister Institute lab assistants' work could be acknowledged in a paper, but they were not allowed to be co-authors ‘because they cannot be regarded as sharing responsibility for the views and conclusions expressed’. Dale added that this had been his personal practice, although his own technician Collison was allowed to publish on technical equipment he invented.^64^ However, he had not imposed the policy generally on the NIMR, and some colleagues made it either a special or regular practice to include technicians as authors, although he did not personally approve of the arrangement.^65^ Mellanby agreed with Dale, although he suggested that the practice not be forbidden entirely and that ‘a certain amount of latitude and discretion [be given] to the qualified worker’.^66^ The very next day Dale circulated a memorandum to all NIMR staff, stressing that for the sake of uniformity of practice throughout the Institute, unqualified assistants who made ‘measurements and manipulations to order’ could not take responsibility for planning research that involved ‘matters of scientific fact or principle’ and therefore could not be included as authors. This he wrote, was an invariable rule, the only exception being the invention of equipment or a technical improvement.^67^

Sometimes technicians were merely at the end of a rather long list of acknowledgements. Sometimes they were simply not acknowledged at all, as Alan Brownstone recalled: ‘It varied a lot, and it was a source of a lot of ill feeling’.^68^ He was one of the first technicians in the NIMR to have his name routinely acknowledged and also by authorship,^69^ although he remembered that ‘[technicians] weren't expected to go to the library; in fact if you saw a brown coat in the Library it was almost a question of, “Oh what do you want? What are you doing here?”’^70^ He was also actively encouraged by his boss, Dr Rosalind Pitt-Rivers, to publish independently, a view that was not always popular with the Director, Sir Charles Harington. Brownstone synthesized an entirely new preparation of deoxyuridine in the early 1960s, which he wrote up for submission to *Nature*. As required by the MRC it was submitted to the Director for approval. Approval was not forthcoming, until finally Pitt-Rivers convinced Harington that Brownstone had done all the work and deserved credit.^71^ An edict was then issued to all staff that technicians' names *could* go at the top of a paper, provided they were able to deliver a talk on the subject. As with much else in the NIMR, it was the arrival of Sir Peter Medawar that heralded enormous changes. Alan Brownstone emphasized that ‘… certainly when Medawar turned up, you found Technicians’ names getting in the top of the paper'.^72^ Another technician elaborated the point:

it got to the eventual stage, which is even before I left, where everybody who participated in a paper got listed at the beginning in alphabetical order … which meant that yours truly, or Alan Brownstone, came at the head, and we got all the correspondence from all the various university libraries to deal with.^73^

## Technical work

Technicians enabled the research work of the NIMR in several ways and at many levels, depending on seniority. A basic distinction is that many worked either for individual, or groups of, scientists in specific departments, whereas others became specialized in providing particular departmental or institute-wide services.

In the former category, Pat Clark joined the parasitology division in 1954 and remained there for 15 years. She recalled the stability that she believed such a length of time provided for the lab:

Technical Staff really were a permanent base there. And there were obviously permanent Scientific Staff—particularly Heads of Division and so on—but most Scientists then used to come and go. The Technical Staff were always there. And so although we didn't particularly know what they were working on, we knew how to run the lab, how to do all sorts of basic things that they would want, and also we knew a lot of people.^74^

Several technicians made more immediate and singular contributions to the design, feasibility and completion of experiments. Jon Marsh recalled his experiences in the same parasitology division, which he joined in mid 1960 to work for Dr Neil Brown on trypanosomiasis.

I was a good Technician. I'm quite a practical bloke, and I think Neil Brown was pretty pleased with what I did, because I could do things that other people couldn't do. It wasn't very long before I was the Starch Gel King. You know, no one else could make these, well there was one other person who could do starch gels.^75^

Starch gel electrophoresis was then a new method for separating protein molecules, and with Brown, Marsh watched the technique in the biological standards division, and then went back to replicate it in his own lab. Starch gels, Marsh emphasized, were

quite tricky things in that you had to warm the starch up in a big round-bottomed flask with the water; and when it got to, somehow you had to know just at the right moment when it was floppy, the right floppiness and you had to de-gas it. And if you didn't de-gas it at the right time, then it didn't de-gas; and it was full of bubbles and it was no use. And it was just a matter of knowing when it was just the right colour and exactly the right feel of floppiness. … I think starch gels were quite good fun, because that's when I was introduced to swearing.^76^

Many technicians developed similar specialized skills, and a ‘feeling’ for their preparations and techniques.^77^ In a slightly different MRC context, that of monoclonal antibody development at the MRC's Molecular Biology Laboratory in Cambridge, the Nobel Laureate Cesar Milstein has described his own technician: ‘Shirley Howe, who was the bearer of the technology over the years, much better of course than I could do. I was a bit of a red fingers, if you like, in contrast to her green fingers.’^78^

Marsh's technical skills were also put to good use in one of the more routine activities of the lab, which was counting trypanosomes in blood smears, as a measure of infectivity.

[A] lot of the work we did was counting cells down the microscope. And how we did it then was, we had these little tally counters—these little grey things with a press-button on top; when you pushed it counted, one, two, three. And you'd be looking down, and you'd be counting a number of fields; and then you'd be saying, ‘How many trypanosomes are there, and you'd say—— I think you had to count two hundred red cells and then how many tryps per two hundred reds: that was the count. And it was a real, actual pain doing this, and we spent a lot of hours a day doing this sort of thing. I don't know why I got involved with this, but I somehow said to Neil, ‘You know what we really want for this is an automatic way of counting these cells; this is such a bind.’ And I think perhaps I wasn't all that busy, so he said, ‘Well ….’ He asked the Electronics Department here if they could build something, and they said they'd think about it. And somehow or other, I can't remember exactly how, I said, ‘Well, I'll go along and build it.’^79^

Marsh's enjoyment of tackling the problem, and his success in constructing such a counter while still a junior technician, were important factors in his decision to transfer into the electronics department, as described below.

Arthur Hemmings recalled several examples of developing experimental techniques and apparatus to enable various members of the scientific staff to perform their biochemical work. One was Dr Don Elliott trying to extract bradykinin, as Hemmings recalled:

I didn't actually work for anybody [because he was then the head technician of the entire division] so when Don Elliott came to me and said ‘I'm going to try and do some work with a large amount of blood, what do you think about tackling it?’ I said well, all the apparatus is here. He hadn't got a clue you see, we had centrifuges in the large-scale lab and things and that and I knew about those.^80^

To do the extraction, Hemmings devised a continuous dialysis apparatus, which did, he added, ‘work out super. But it was never published.’^81^ Similarly, it was his ingenuity that was called into play by (Sir) Frank Young when working on the extraction of pituitary factors. Because of the large volume of material required to obtain even small amounts of precipitate, whale pituitaries were obtained. Hemmings devised an extraction procedure, using a Sharples continuous-flow centrifuge, with a feed tube at a height of 8 feet, in a cold room to reduce the deterioration of the end precipitate, and with continuous recycling of the pituitary material, to achieve maximum extraction. It was to be a laborious and difficult technique. Hemmings described the night before the experiment, ‘There were about ten 25-litre buckets at least half full [of whale pituitary], I said that's all right, get in early [tomorrow], we'll get started. I thought he [Young] was going to help me. I got in next morning about 8 a.m.’ Hemmings started the experiment, running up and down ladders with heavy buckets of increasingly macerated pituitary to keep the continuous equipment running. He saw nothing of Young. ‘At about one o'clock’, Hemmings recalled, ‘Young was going out to lunch obviously, he looked in and enquired how I was doing. I was livid, I didn't like being treated like that’.^82^

Perhaps because of such experiences, others deliberately chose to work in a service department rather than have close involvement with particular strands of research. John Clark, who started at the NIMR in 1952, has described his motives:

I suppose I felt that I would feel better about working a life-time as it were if I had what I thought of then as a sort of trade, rather than being an academic sort of scholar-type person. I would have a skill, which these high-up people couldn't actually do. And that attracted me; so I went in for— I'd always been interested in photography at the time, and I was particularly attracted to microscopy. So I went into the Department of Biophysics and Optics, as it was called then, under someone called Mr Smiles, who was a Scientist, and Mr Young, Robert Young, who was Head Technician, I suppose you'd call him. And there was me, and a couple of other people. Now [speaking in 1998] that sort of job is just an adjunct of the Medical Illustration Department. But then it was a sizeable Section, if you know what I mean. It was right in the middle of the exploration of phase-contrast microscopy. It was the tail-end of the use of UV microscopy, and the very beginning, well not long after I went there, of fluorescence microscopy. … As I say, it gave you a skill, in which you were some service to all sorts of people. The other thing I liked about it was that you worked for lots of different people.^83^

Clark transferred to the MRC's Clinical Research Centre at Northwick Park as head technician 20 years later; he was then responsible for all photomicrography (light and electron) throughout the Centre. Others have also commented on the diversity of contacts that those in the service departments enjoyed. After five years in parasitology and with a taste for technical challenges after his development of the automatic cell counter, Jon Marsh moved into the engineering division to retrain to provide electronics support. ‘As a service function’, he said, ‘we had frequent contact with most labs except perhaps those concentrating mainly on chemistry’.^84^ By the time he retired in the late 1990s, he was head of that Division, and responsible for all engineering and routine equipment servicing throughout the NIMR, and all computer support.

## Conclusion

This study, focusing on the technical staff of the NIMR, has examined how, in the early years of the twentieth century, as medical laboratories were being created, then so too were new categories of staff—dedicated research workers and ancillary support staff. Initially, support staff were employed almost as domestic servants and were usually young boys who fetched, carried, polished and mended, doing as one of the interviewees here put it, ‘[E]verything’.^85^ The changing demands of medical research, as questions and methodology became more specialized, meant that rarely did one person have the necessary theoretical and technical expertise to perform an extensive piece of research on their own, and collaborations and research teams of two or more people with different abilities and interests became increasingly usual. Thus, ‘lab boys’ who improved their manual and intellectual skills, either at work or more usually through evening classes, became identified as ‘lab assistants’, and their importance was increasingly recognized by an expansion in the number of technical posts and improved terms and conditions of service. However, the word ‘technician’ was not routinely used until World War II, and it was during that conflict, both at the front and at home, that the value of technical work was widely acknowledged. At the same time, social and professional divisions between technicians and scientists started to break down, and with postwar reconstruction and union representation came an increasing tendency for technicians to work with (rather than for) scientists, to get their contributions explicitly acknowledged, and to benefit from day-release and other training courses. Their longevity in post, at a time when scientific staff moved quite frequently, often provided a consistency and stability that maintained the very culture of the laboratory. By the 1960s laboratory technicians were no longer completely invisible, but they remain difficult to see, and the difficulties of tracing and then recording the experiences of technicians remain major obstacles. This paper has therefore attempted to remedy some of those deficits, by using extensive oral history interviews, and to begin a narrative and analytical account of what constituted technical work in twentieth-century British medical research.
